# Analysis of sequencing strategies and tools for taxonomic annotation: Defining standards for progressive metagenomics

**DOI:** 10.1038/s41598-018-30515-5

**Published:** 2018-08-13

**Authors:** Alejandra Escobar-Zepeda, Elizabeth Ernestina Godoy-Lozano, Luciana Raggi, Lorenzo Segovia, Enrique Merino, Rosa María Gutiérrez-Rios, Katy Juarez, Alexei F. Licea-Navarro, Liliana Pardo-Lopez, Alejandro Sanchez-Flores

**Affiliations:** 1 Consorcio de Investigación del Golfo de México (CIGOM), Instituto de Biotecnología, Universidad Nacional Autónoma de México, Cuernvaca, Mexico; 2 Instituto de Biotecnología, Universidad Nacional Autónoma de México, Cuernvaca, Mexico; 3 Departamento de Innovación Biomédica, CICESE. Carretera Ensenada-Tijuana 3918, Zona Playitas, Ensenada, BC Mexico

## Abstract

Metagenomics research has recently thrived due to DNA sequencing technologies improvement, driving the emergence of new analysis tools and the growth of taxonomic databases. However, there is no all-purpose strategy that can guarantee the best result for a given project and there are several combinations of software, parameters and databases that can be tested. Therefore, we performed an impartial comparison, using statistical measures of classification for eight bioinformatic tools and four taxonomic databases, defining a benchmark framework to evaluate each tool in a standardized context. Using *in silico* simulated data for 16S rRNA amplicons and whole metagenome shotgun data, we compared the results from different software and database combinations to detect biases related to algorithms or database annotation. Using our benchmark framework, researchers can define cut-off values to evaluate the expected error rate and coverage for their results, regardless the score used by each software. A quick guide to select the best tool, all datasets and scripts to reproduce our results and benchmark any new method are available at https://github.com/Ales-ibt/Metagenomic-benchmark. Finally, we stress out the importance of gold standards, database curation and manual inspection of taxonomic profiling results, for a better and more accurate microbial diversity description.

## Introduction

For decades, important advances in microbial ecology and many other fields, have been achieved thanks to the possibility of studying microbial communities by characterizing their genetic information. While the 16S rRNA gene has been widely accepted as a biological fingerprint for bacterial species, it presents some limitations. Many bacterial species have multiple 16S rRNA gene copies, leading to an artificial diversity overrepresentation^1^. Between some bacterial species, there are no significant differences in their 16S rRNA genes, but other genomic elements will confer them important features that will differentiate them as pathogens or harmless free-living organisms^2,3^. Other technical considerations regarding the characterization of the 16S rRNA gene, are primer and amplification biases^4^, chimera formation^4,5^ and other artifacts that make difficult the assessment of the real community structure, like the microheterogeneity of sequences between closely related strains, or the similarity of sequences between non-closely related species.

The use of high-throughput sequencing technologies has allowed the analysis of very complex environmental samples either by 16S rRNA gene amplification or Whole Metagenome Shotgun (WMS) sequencing which could retrieve the genomic information from all the organisms present in the sample. Also, bioinformatics tools have been redesigned to cope with the massive amount of data generated by high-throughput sequencing technologies. Advantages and limitations of sequencing strategies and metagenomic analysis software have been vastly described before^4,6–11^. However, the selection of sequencing or bioinformatic approaches for any project, remains a challenge due to several factors such as the constant change of sequencing technologies, database updating and rapid software development.

Arguably, the biggest challenge is the reduction of introduced biases in metagenomic studies. Sample handling and preservation^12–14^; DNA extraction technical issues^15^; sequencing technology artifacts^6,10,16^ and bioinformatic analysis limitations^17^ contribute to analysis biases.

To understand these problems and to elucidate the origin of different biases in a real sample, it is necessary to analyze the contribution of individual variables to a certain bias. These biases could be reflected either in an over- or underestimation of diversity depending on the sample handling variables and software parameters used in the sequences analysis. There are few reference datasets^18–20^ which can be used as a gold standard for every metagenomic project, allowing the control of different variables to evaluate tools impartially. Researchers have to select one of the many available tools or develop a new one to analyze their metagenomic data. Usually, even if a benchmark is performed comparing different tools, authors often use distinct metrics to evaluate the method performance. Also, benchmark results will vary if databases change or the software parameters or version change.

Here, we performed an objective comparison based on performance statistical measures of classification and error rate at different taxonomic levels. We used three *in silico* datasets for WMS and V3-V4 16S rRNA amplicon Illumina simulated reads, to evaluate eight different bioinformatic tools and seven public databases. We standardize the taxonomic annotation lineage by correcting all results based on the NCBI taxonomy database. Additionally, we report coverages and cut-off score values at different error rates for all tested methods.

Our goal is to contribute to standards and metric definition for metagenomic analysis through a standardized benchmark framework to constantly evaluate sequencing strategies, taxonomic profiling tools and databases.

## Materials and Methods

In this work, we chose a set of tools, used for taxonomic annotation of metagenomic samples, that could be installed in a local computer server. We used a 64 core/512 Gb of RAM PC server, using Ubuntu 16.04 Linux distribution, to perform all of the present work.

The performance of each program was evaluated with *in silico* sequences generated to simulate Illumina reads for whole metagenome shotgun (WMS) and amplicons from the V3-V4 variable region of 16S rRNA gene, in triplicate. We estimated, through error type and coverage calculation, the bias due to either the algorithm or the database used at different taxonomic levels from phylum to subspecies.

### In silico datasets for WMS and 16S rRNA profiling

Datasets for WMS analysis were obtained from the data published by Lindgreen *et al*.^17^ In order to obtain triplicate information, we choose the A1, A2 and A3 datasets which originally contained bacterial, archaea and eukarya genomes. However, in order to delimit our analysis, we removed eukaryotic genomes since the evaluated programs were not designed to evaluate eukaryotic information. Each dataset (A1, 673 genomes; A2, 678 genomes and A3, 674 genomes) had *in silico* simulated paired-end sequences of 100 bp length for each species genome. Lindgreen datasets also include divergent “shuffled” sequences from some species that are not supposed to be annotated (true negatives) and simulated reads with variable evolutionary distances generated by phylogenetic modelling to mimic nonexistent close relatives from *Leptospira interrogans* genome, which are expected to be classified somewhere on the *Leptospira* taxonomic lineage but not necessarily at genus or species levels. Sequences can be found at http://www.ucbioinformatics.org/metabenchmark.html.

To evaluate the performance of each program with amplicon datasets, we generated three amplicon libraries from V3-V4 variable regions of ribosomal 16S rRNA gene using the Grinder v0.5.4 software^21^. For *in silico* PCR we used primers S-D-Bact-0341-b-S-17 and S-D-Bact-0785-a-A-21^22^ and, as template, we extract the 16 S ribosomal sequences from the gbk files of the 840 bacterial genomes used by Lindgreen *et al*.^17^. Amplicon libraries shared the 90% of reference sequences and were constructed simulating 750,000 paired-end reads of 300 bp length using a linear abundance model and a per-base quality fixed in 30 Phred score. Additionally, we include in each library a set of 37,500 unclassifiable homemade shuffled sequences to assess as true negatives. The amplicons were rebuilt by Flash v1.2.11^23^ and extended fragments were used to perform the taxonomic annotation. Sequences are available at https://github.com/Ales-ibt/Metagenomic-benchmark.

### Taxonomic classification software

Four open source bioinformatic tools for WMS data^24–27^ and four different software for amplicon sequences^28–31^ were tested. In the particular case of Kraken and CLARK, specific databases based on k-mer spectra from RefSeq genomes were used. Uclust algorithm was used for clustering in QIIME pipeline as it is the default option. All methods based on ribosomal sequences annotation were tested using the main databases publicly available: Ribosomal Database Project (RDP) v11.5^32^ available at https://rdp.cme.msu.edu/misc/resources.jsp; SILVA v128^33^ can be downloaded from https://www.arb-silva.de/no_cache/download/archive/release_128/; GreenGenes (GG) v13.5^34^ from http://greengenes.secondgenome.com/downloads/database/13_5 as well as Metaxa2 database (MTX)^31^ that is included in the Metaxa2 software package. The database version could change if the program includes its own database with the software distribution as in the case of Parallel-meta. Specifications about the software tested are described in Supplementary Table 1.

### Software performance evaluation

To evaluate the different methods of analysis for both amplicons and WMS, we performed a binary classification test of TRUE or FALSE assignments per read comparing the taxid of the expected lineage against the taxid of the taxonomic annotation at every taxonomic level (domain, phylum, class, order, family, genus, species and subspecies). The TRUE ratings could be due to a correct taxonomic identification, i.e. a true positive annotation (TP); or a non-classification of a “shuffled” sequence, i.e. a true negative (TN). A FALSE classification means a misclassification that implies an erroneous annotation, i.e. a false positive (FP); or a non-classification at an specific taxonomic level, that means a false negative (FN). If we have a correct booked assignment up to family level, the table for this read looks like: TP, TP, TP, TP, TP, FN, FN, FN. However, in the case of a correct annotation to family level but erroneous genus level, we fill the table with TP, TP, TP, TP, TP, FP, FP, FP. Therefore, we are capable to differentiate between non-classification and misclassification at each taxonomic level. In the case of WMS data, the universe of classifiable sequences depends on the annotator approach (reads comparison with phylogenetic markers or k-mer spectra), The selection of the taxonomically informative sequences, depends on the extraction algorithm and is different for each method. Nevertheless, the above error definition works the same for amplicon and WMS data.

With this information, we built a confusion matrix from which we calculated performance statistical measures of classification such as sensitivity, specificity, and accuracy. Additionally, we used the Matthews Correlation Coefficient (MCC) as global description of the confusion matrices but weighing the compared classes (true or false positive and negatives). Values of MCC equal to zero, indicates that a tested combination generated results as good as obtaining them by random; a negative MCC score indicates results worse than obtaining them by random^35^. Formula used to calculate each descriptor are below:

$$\begin{array}{c}{\bf{E}}{\bf{P}}{\bf{Q}}\,({\bf{E}}{\bf{r}}{\bf{r}}{\bf{o}}{\bf{r}}\,{\bf{P}}{\bf{e}}{\bf{r}}\,{\bf{Q}}{\bf{u}}{\bf{e}}{\bf{r}}{\bf{y}})={\bf{F}}{\bf{P}}/{\bf{T}}{\bf{o}}{\bf{t}}{\bf{a}}{\bf{l}}\,{\bf{q}}{\bf{u}}{\bf{e}}{\bf{r}}{\bf{y}}\,{\bf{n}}{\bf{u}}{\bf{m}}{\bf{b}}{\bf{e}}{\bf{r}}\\ {\bf{C}}{\bf{o}}{\bf{v}}{\bf{e}}{\bf{r}}{\bf{a}}{\bf{g}}{\bf{e}}\,({\bf{C}}{\bf{o}}{\bf{v}})={\bf{T}}{\bf{P}}/{\bf{T}}{\bf{o}}{\bf{t}}{\bf{a}}{\bf{l}}\,{\bf{e}}{\bf{x}}{\bf{p}}{\bf{e}}{\bf{c}}{\bf{t}}{\bf{e}}{\bf{d}}\,{\bf{r}}{\bf{e}}{\bf{s}}{\bf{u}}{\bf{l}}{\bf{t}}{\bf{s}}\,{\bf{n}}{\bf{u}}{\bf{m}}{\bf{b}}{\bf{e}}{\bf{r}}\\ {\bf{S}}{\bf{e}}{\bf{n}}{\bf{s}}{\bf{i}}{\bf{t}}{\bf{i}}{\bf{v}}{\bf{i}}{\bf{t}}{\bf{y}}\,({\bf{a}}.{\bf{k}}.{\bf{a}}\,{\bf{T}}{\bf{r}}{\bf{u}}{\bf{e}}\,{\bf{P}}{\bf{o}}{\bf{s}}{\bf{i}}{\bf{t}}{\bf{i}}{\bf{v}}{\bf{e}}\,{\bf{R}}{\bf{a}}{\bf{t}}{\bf{e}}\,{\bf{o}}{\bf{r}}\,{\bf{R}}{\bf{e}}{\bf{c}}{\bf{a}}{\bf{l}}{\bf{l}})={\bf{T}}{\bf{P}}/({\bf{T}}{\bf{P}}+{\bf{F}}{\bf{N}})\\ {\bf{S}}{\bf{p}}{\bf{e}}{\bf{c}}{\bf{i}}{\bf{f}}{\bf{i}}{\bf{c}}{\bf{i}}{\bf{t}}{\bf{y}}\,({\bf{a}}.{\bf{k}}.{\bf{a}}\,{\bf{T}}{\bf{r}}{\bf{u}}{\bf{e}}\,{\bf{N}}{\bf{e}}{\bf{g}}{\bf{a}}{\bf{t}}{\bf{i}}{\bf{v}}{\bf{e}}\,{\bf{R}}{\bf{a}}{\bf{t}}{\bf{e}})={\bf{T}}{\bf{N}}/({\bf{T}}{\bf{N}}+{\bf{F}}{\bf{P}})\\ {\bf{A}}{\bf{c}}{\bf{c}}{\bf{u}}{\bf{r}}{\bf{a}}{\bf{c}}{\bf{y}}\,({\bf{A}}{\bf{C}}{\bf{C}})=({\bf{T}}{\bf{P}}+{\bf{T}}{\bf{N}})/({\bf{T}}{\bf{P}}+{\bf{F}}{\bf{P}}+{\bf{F}}{\bf{N}}+{\bf{T}}{\bf{N}})\\ {\bf{M}}{\bf{a}}{\bf{t}}{\bf{t}}{\bf{h}}{\bf{e}}{\bf{w}}{\bf{s}}\,{\bf{C}}{\bf{o}}{\bf{r}}{\bf{r}}{\bf{e}}{\bf{l}}{\bf{a}}{\bf{t}}{\bf{i}}{\bf{o}}{\bf{n}}\,{\bf{C}}{\bf{o}}{\bf{e}}{\bf{f}}{\bf{f}}{\bf{i}}{\bf{c}}{\bf{i}}{\bf{e}}{\bf{n}}{\bf{t}}\,({\bf{M}}{\bf{C}}{\bf{C}})\,=\\ \,({\bf{T}}{\bf{P}}\ast {\bf{T}}{\bf{N}})-({\bf{F}}{\bf{P}}\ast {\bf{F}}{\bf{N}})/{[({\bf{T}}{\bf{P}}+{\bf{F}}{\bf{P}})({\bf{T}}{\bf{P}}+{\bf{F}}{\bf{N}})({\bf{T}}{\bf{N}}+{\bf{F}}{\bf{P}})({\bf{T}}{\bf{N}}+{\bf{F}}{\bf{N}})]}^{{\bf{1}}/{\bf{2}}}\end{array}$$

### Coverage versus error per query plots generation

We generated coverage versus error or CVEs plots. Sequences assigned by each method were ordered from best to worst according to the respective reported score, then, we summed the number of false positives in the total number of queries to obtain the Error per query (EPQ) and we plotted it against the number of true positives divided by the total number of expected results (Coverage)^36–38^. The CVE plots for each taxonomic level were elaborated using the R software^39^. Such graphical representation allows visualizing directly the error accumulation as a function of the proportion of sequences annotated at each taxonomic level, without needing to observe the areas under the curve. Besides, it is possible to obtain the score cut-off value where each method reaches a given error value (see Supplementary Table 2).

Each method reports a particular assignment score. In the case of Kraken, we used the k-mers percentage of allocation with respect to the reference that the program assigns for each read, in order to establish a classification score for each assignment. CLARK reports a confidence score. The ranking for Parallel-Meta was the E-value, for Metaxa2 was the reliability score and for MetaPhlAn2 and MOCAT the alignment score was taken. In the particular case of SPINGO, we rank the similarity score in the output file and in the cases where the annotation was found as AMBIGUOUS, we extracted the lowest common ancestor (LCA) from the list of reported species. All plots are available at https://github.com/Ales-ibt/Metagenomic-benchmark

### Taxonomy lineage homogenization

In order to homogenize the assignments for each method and to determine the complete lineage adjusting to fill the eight basic ranks: domain, phylum, class, order, family, genus, species, and subspecies, we used the taxid according to NCBI Taxonomy database and we parsed the information by ETE 3 python library^40^. The process for obtaining an integrated matrix of all methods per sample required a set of scripts written in R, bash, perl and python, which are available at https://github.com/Ales-ibt/Metagenomic-benchmark

## Results

### Score equivalence by error rate using CVE plots

We evaluated eight different methods to determine the relation between sensitivity and specificity for each tool/database combination at eight taxonomic classification levels, using simulated data for either amplicons from 16S rRNA V3-V4 regions or Whole Metagenome Shotgun (WMS) reads. Some methods were combined with different databases but others only worked using their own database (see Materials and Methods). To compare results from different methods where each one uses a different score value, we used Coverage VS Error per query (CVE) plots (available from https://github.com/Ales-ibt/Metagenomic-benchmark) to visualize the error rate and coverage associated with different score values. A better method would depict a graph with a lower slope, meaning a higher coverage at a lower error rate.

To address the great volume of generated results, we presented them in subsections from an algorithm perspective. We evaluated tools by classifying them in BLAST-alignment and BLAST-independent based methods for 16S rRNA amplicon or Whole Metagenome Shotgun data. The cut-off score, coverage and standard deviation values for each method at 1%, 5% and 10% error rate at the eight different taxonomic classification levels, can be found in Supplementary Table 1.

### BLAST-alignment based methods using 16S rRNA amplicon sequencing

**Figure 1:**
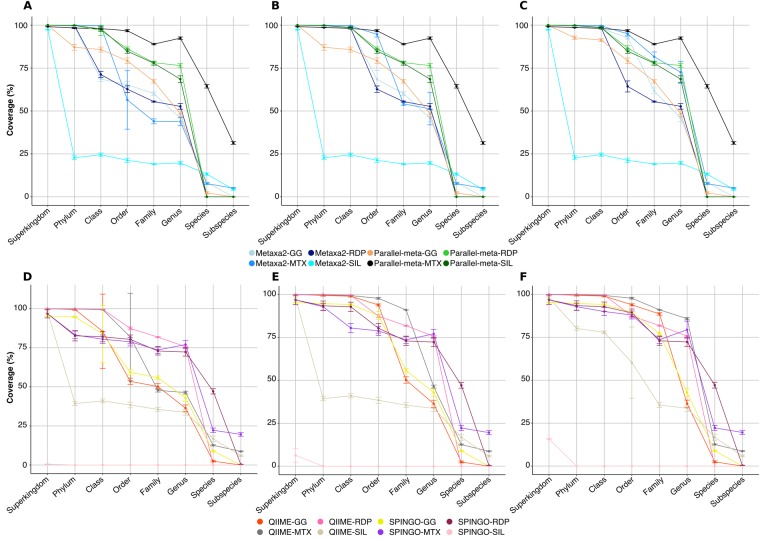
Maximum coverage reached at each taxonomic level for methods tested in 16S rRNA amplicon datasets. Panels from A-C corresponds to BLAST-alignment based methods and represents coverage at (**A**) 1%, (**B**) 5%, (**C**) 10% error cut-offs. Panels from (**D**–**F**) corresponds to BLAST-independent based methods and represents coverage at (**A**) 1%, (**B**) 5%, (**C**) 10% error cut-offs. The main differences are observed at class and order taxonomic levels for Metaxa2, SPINGO-GG and QIIME-GG methods.

We evaluated a modified version of Parallel-meta v2.4.1 (Material and Methods) and Metaxa2 v2.1.1, in combination with four different databases. As mentioned, we generated the CVEs plots at eight taxonomic levels. To observe the performance of each tool/database combination, we summarize in Fig. 1A–C the coverage results only at 1,5 and 10% error cut-off values, for all taxonomic levels.

At higher taxonomic levels (domain and phylum), Parallel-meta and Metaxa2 combinations, reported the highest coverage (>95%) even at the lowest error rate (1%). In the case of Metaxa2-SILVA combination, the coverage dropped below 25% at phylum level being the lowest value of all method/database combinations and error rates (Fig. 1A–C). In a similar trend but with a less drastic drop, Parallel-meta-GG presented lower coverage values (<90%) at phylum level, at 1 and 5% error rate (Fig. 1A,B).

However, at intermediate taxonomic levels (class to family) the only method with coverage greater than 75% and a cumulative error equal or less than 1%, was Parallel-meta in combination with SILVA, RDP and MTX databases (Fig. 1A). Interestingly, at the genus rank, the only tool-database combination that presented over ~87% of expected coverage at 1% error rate, was Parallel-meta-MTX and for this combination, at species and subspecies levels, the coverage at 1% error was the highest among all combinations.

**Figure 2:**
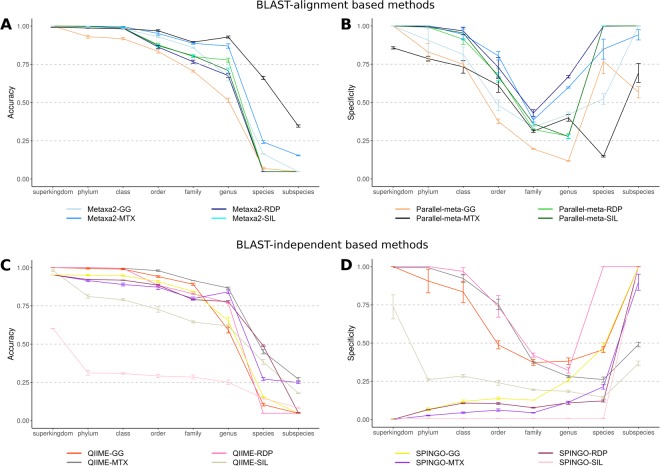
Performance descriptors plots calculated for methods tested in 16S rRNA amplicon datasets annotation. Panels A and B corresponds to accuracy and specificity for BLAST-alignment based methods. Panels C and D corresponds to accuracy and specificity for BLAST-independent based methods. The score scale from 0 to 1 correspond the order of inner to outer circles.

To evaluate further, other metrics such as accuracy and specificity were used to evaluate the performance of each combination (Fig. 2A,B). Parallel-meta-GG had the lowest accuracy, even at phylum level, in contrast to Parallel-meta-MTX which presented the highest accuracy at all taxonomic levels. At the genus rank, Parallel-meta-MTX reached an accuracy of 93% followed by Metaxa2-MTX with an accuracy of 86% (Fig. 2A).

In terms of specificity, all methods presented low values at different taxonomic levels. In general, methods based on local alignment algorithms (BLAST), had a high true positives rate but also a high false positive rate. The lowest specificity values were observed at the family level, where all methods had a high number of false positives. At genus, species and subspecies ranks, the true negative rate increased gradually (Fig. 2B), consistently with the accuracy drop at these same taxonomic levels (Fig. 2A).

### BLAST-independent based methods using 16S rRNA amplicon sequencing

We tested QIIME v1.9.1 and SPINGO v1.3 programs using the same datasets and database combinatorial design than described above. We found that SPINGO neither performed well with SILVA nor with GreenGenes databases, but in combination with MTX or RDP databases had a better performance (Fig. 1D–F).

The method with the lowest performance between phylum and family taxonomic levels was QIIME-SILVA, with coverage values from 30 to 45% at 1% of error rate (Fig. 1D). At the same error rate, only QIIME using either RDP or MTX databases, presented coverage values higher than 90% at domain, phylum and class taxonomic levels. Both SPINGO and QIIME in combination with GG database, presented the highest result variation among replicates. At family and genus levels, both SPINGO-RDP, QIIME-RDP and SPINGO-MTX combinations, performed very similarly maintaining coverages above ~75%. Finally, at the genus level, both methods underperformed when combined with GG and SILVA databases (Fig. 1D).

The method with the best performance at less stringent error cut-off values (5% and 10%) was QIIME-MTX at the family and genus levels. (Fig. 1E,F). However, at the species level, the accuracy of QIIME-MTX dropped to values under 50%, similar to SPINGO-RDP combination. In general, both methods lost accuracy in their predictions in combination with SILVA database and both tools presented the highest accuracy at genus level in combination with MTX database (Fig. 2C).

### BLAST-alignment based methods using Whole Metagenome Shotgun data

**Figure 3:**
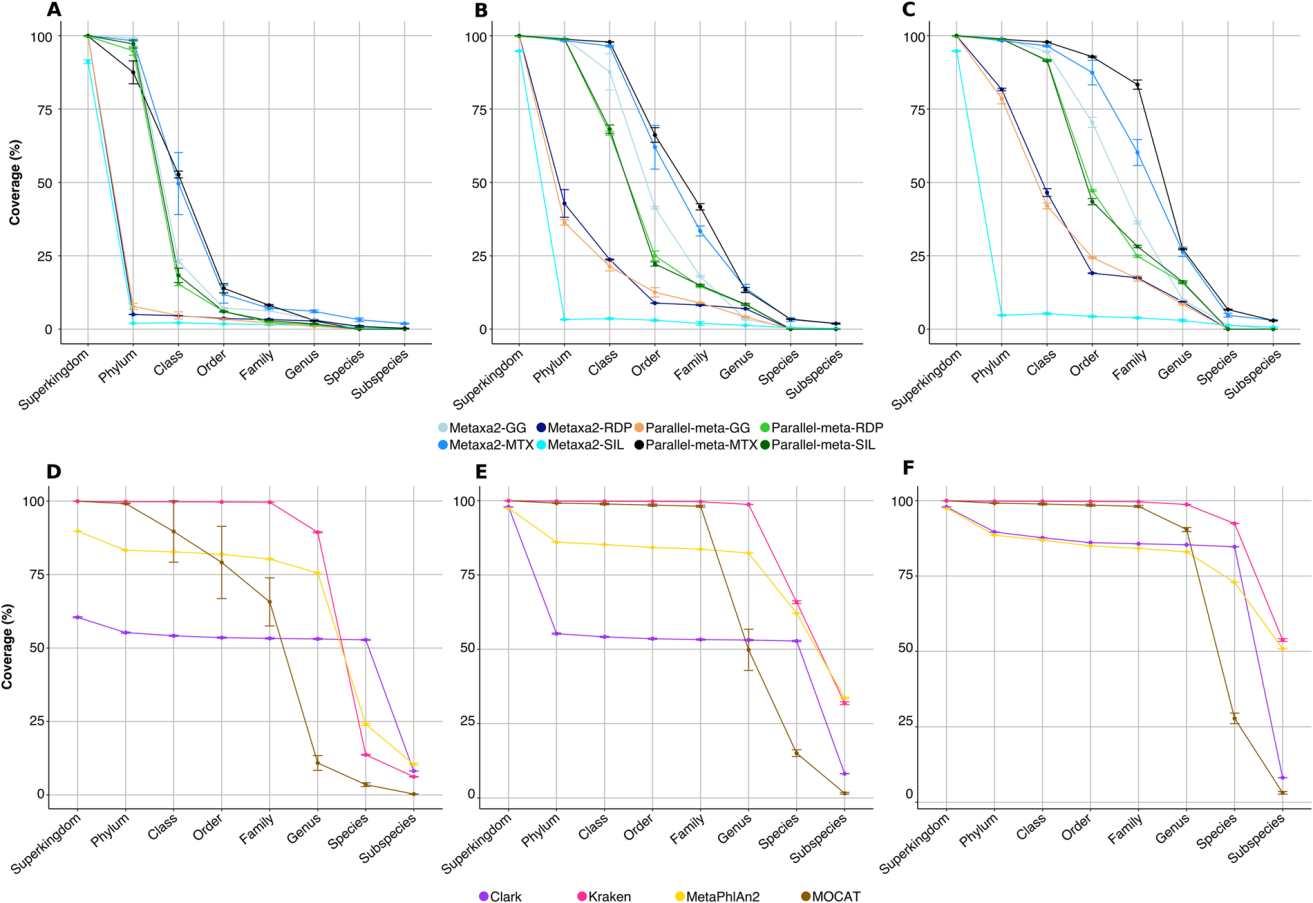
Maximum coverage reached at each taxonomic level for methods tested in whole metagenome shotgun datasets. Panels A-C corresponds to BLAST-alignment based methods and represents coverage at (**A**) 1%, (**B**) 5%, (**C**) 10% error cut-offs. Panels (**D**–**F**) corresponds to BLAST-independent based methods and represents coverage at (**A**) 1%, (**B**) 5%, (**C**) 10% error cut-offs. A coverage decrement is clear from class to family level in all the Blast-alignment based methods (**A**–**C**); Clark showed the lower coverages at 1 and 5% of error thresholds (**D**,**E**).

We evaluated the taxonomic annotation results by the same methods but using the extraction of 16S rRNA sequences from WMS data using Parallel-meta v2.4.1 and Metaxa2 v2.1.1. As depicted in the CVE plots and their summary in Fig. 3A–C, all combinations presented a drastic coverage droppage from class to family taxonomic levels at 1% of error rate. Some combinations like Metaxa2-SILVA, Metaxa2-RDP and Parallel-meta-GG had the lowest performance at any error rate (Fig. 3A–C). At higher error rates (5 and 10%) the methods reported the highest coverage values at class, order and family levels but only in combination with the MTX database (Fig. 3B,C).

**Figure 4:**
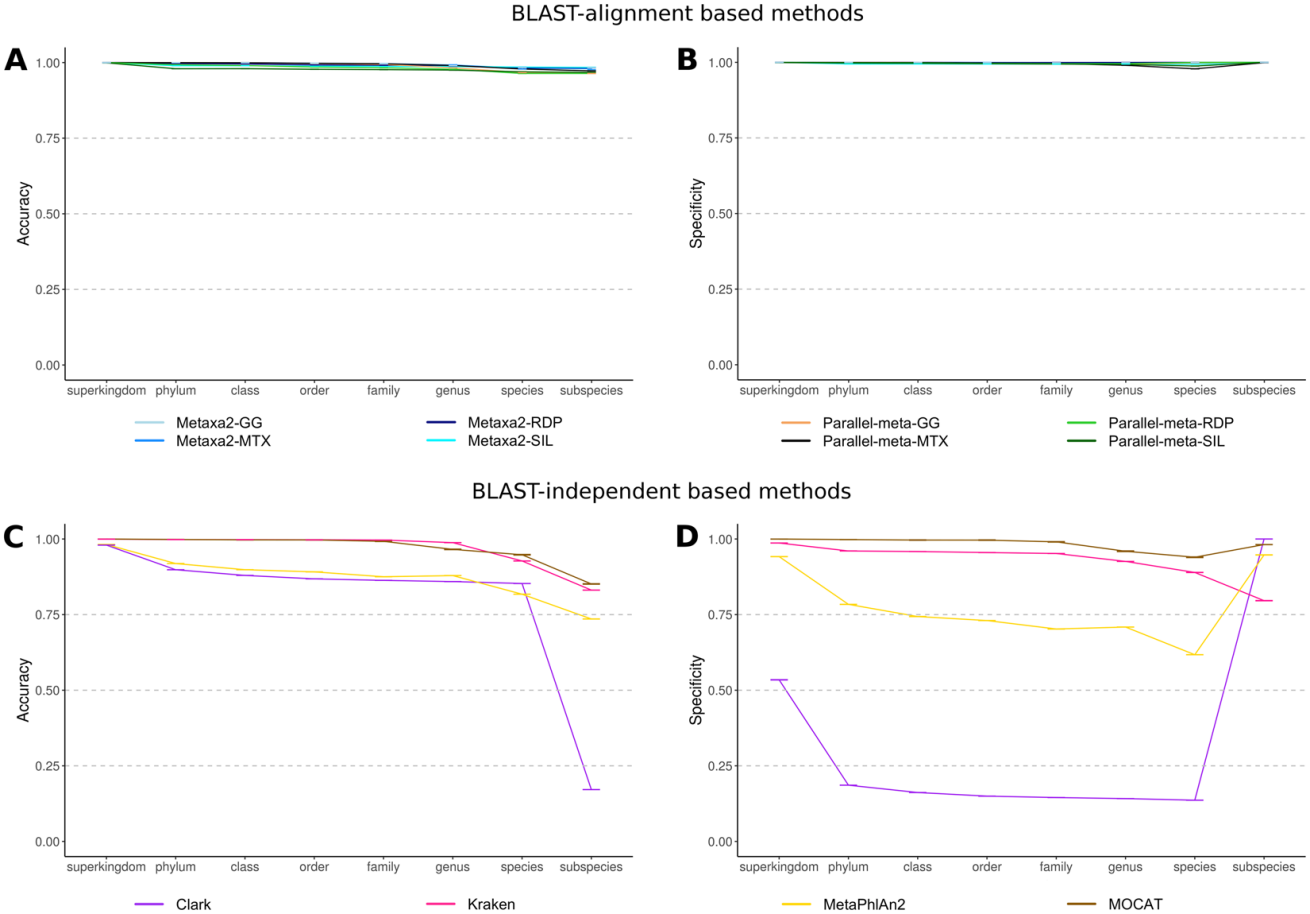
Performance descriptors plots calculated for methods tested in whole metagenome shotgun datasets annotation. Panels A and B corresponds to accuracy and specificity for BLAST-alignment based methods. Panels C and D corresponds to accuracy and specificity for BLAST-independent based methods. The score scale from 0 to 1 correspond the order of inner to outer circles.

Despite the observations in CVE plots, in terms of accuracy and specificity, all methods presented high values at all taxonomic levels (Fig. 4A,B). The difference between methods were observed in terms of sensitivity. The only method with the highest sensitivity from domain to species level was Parallel-meta-MTX. Actually, both methods combined with the MTX database and with GG showed results with a sensitivity >0.75 (up to family level). In contrast, the annotations of both methods in combination with the SILVA database, had the lowest sensitivity even at phylum level (Supplementary Fig. S3).

### BLAST-independent based methods using WMS data

Methods that do not rely solely on the taxonomic information from the 16S rRNA gene, are described in this section. Two of the most popular methods based on k-mer spectra comparison, Kraken v0.10.5-beta and CLARK v1.2.3.1, were used to annotate the WMS datasets. We also analyzed two annotation methods based on single copy marker genes (SCMG), MetaPhlAn2 and MOCAT. Each SCMG method can be used only with its own database; therefore, those results have no database combinations.

MetaPhlAn2 v2.2.0 and Kraken v1.3 reported the highest coverage until genus taxonomic level (75.5% and ~89.4%, respectively) at 1% of error rate. MOCAT showed a coverage drop to ~65% and ~11% at family and genus levels, respectively. We observed an interesting trend for CLARK results, which showed a constant coverage between 60–50% from domain to species taxonomic levels. At species level, CLARK had the highest coverage in comparison to all other methods at 1% of error rate (Fig. 3D).

Using more relaxed cut-off values of 5 and 10%, Kraken and MetaPhlAn2 coverage values at species level were improved. MOCAT showed a coverage value of ~98% up to family level, while CLARK remained with a constant coverage of ~60% from phylum to species (Fig. 3E,F). In general, at 10% error rate, all methods were capable of reporting coverage values above 80% until genus taxonomic level. At species level, MOCAT was the only method with a coverage drop below 30%, while the other methods kept values over 70%. K-mer based methods presented the highest coverage values until species taxonomic level at 5 and 10% of error rate. In terms of accuracy, Kraken and MOCAT performed better than CLARK and MetaPhlAn2 at all taxonomic levels (Fig. 4C). An abrupt accuracy decrease was observed in CLARK (below 25%) at subspecies levels.

The methods with lower false negative rate were MOCAT and Kraken, which maintained specificity values higher than 90% at all taxonomic levels. MetaPhlAn2 maintained values closer to 75% from phylum to genus taxonomic levels. The method with the lowest specificity was CLARK (Fig. 4D).

### Observed biases at phylum level and cut-off error filtering

Since we found that methods can present errors even at higher taxonomic levels such as phylum, we determined if the false positive and false negative (type I and II) errors were distributed evenly among different phyla or had a specific phyla distribution.

**Figure 5:**
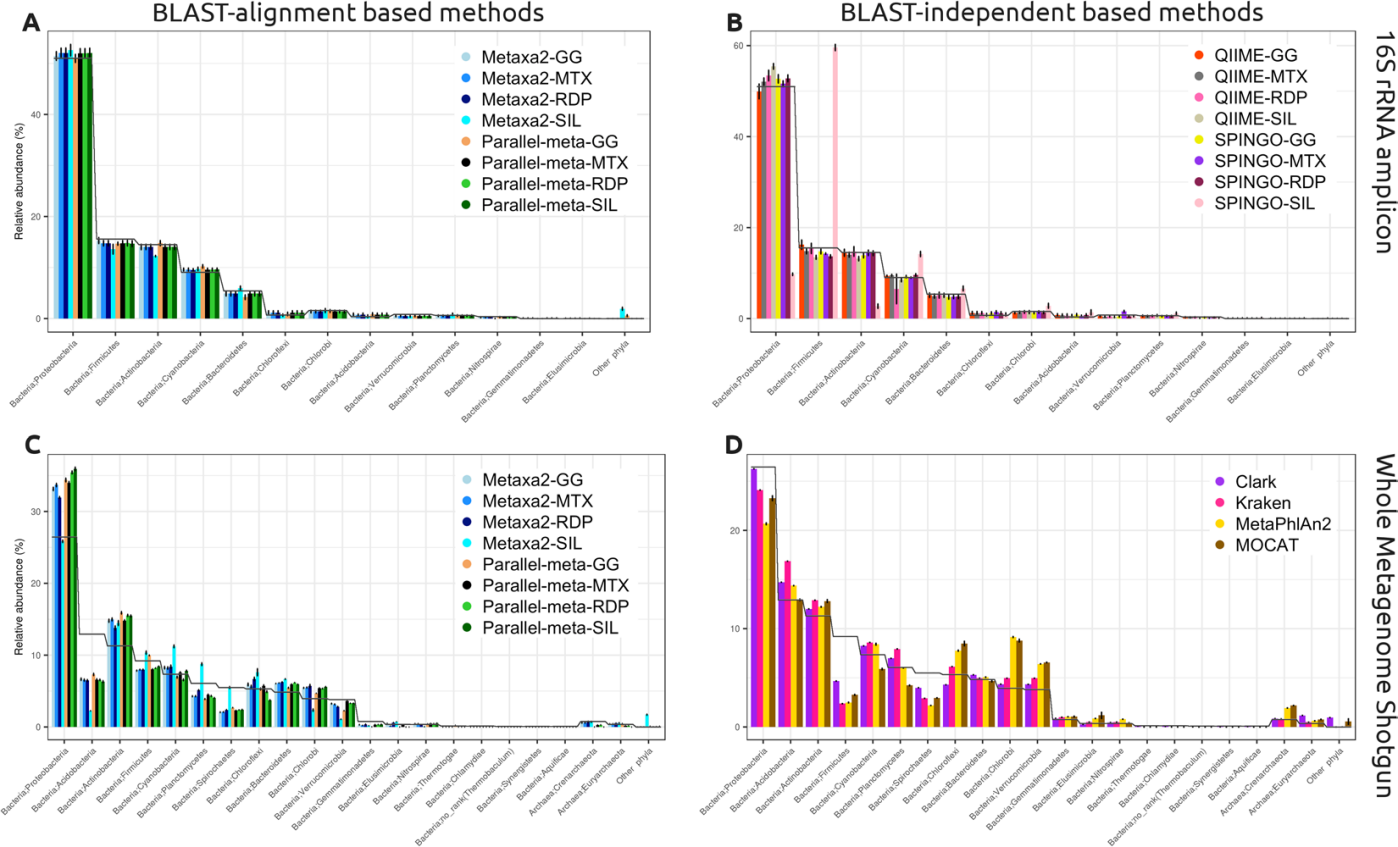
Taxonomic abundance of annotation at phylum level. (**A**) BLAST-alignment based methods on 16S rRNA amplicon data, (**B**) BLAST-independent methods on 16S rRNA amplicon data, (**C**) BLAST-based methods on whole metagenome shotgun data, (**D**) BLAST-independent methods on whole metagenome shotgun data. The black line represents the average of the expected abundance for each plot.

For ribosomal amplicon data results, all BLAST-alignment based methods reported very similar abundances at the phylum level without any remarkable biases (Fig. 5A). Metaxa2-SILVA and Parallel-meta-GG combinations presented different abundances than expected, the former presented false positive results referring to an unidentified_marine_bacterioplankton, while the latter had false positive results referring to Tenericutes and Thermotogae phyla (see Supplementary Table 2).

**Figure 6:**
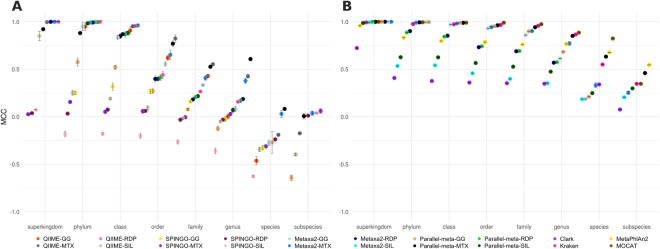
Matthews correlation coefficient (MCC) for (**A**) 16S rRNA amplicon data confusion matrices and (**B**) whole metagenome shotgun data confusion matrices.

BLAST-independent methods showed a similar trend near to the expected abundance of the evaluated data. However, the SPINGO-SILVA combination overestimated the Firmicutes, Cyanobacteria, Bacteroidetes and Chlorobi phyla. This combination also underestimated the Proteobacteria phyla. The method with the greater bias at phyla taxonomic level was SPINGO in combination with GG, MTX and RDP databases according to MCC (Fig. 6A). These combinations presented false positive results distributed in up to 28 different phyla (collapsed in other phyla category in Fig. 5B), although in very low abundance.

In contrast to the results obtained in the taxonomic annotation of ribosomal amplicon sequences, other but more evident biases were observed in WMS data phyla abundances. In general, Metaxa2 and Parallel-meta in combination with most databases, showed a very similar trend and bias. The most evident errors occurred due to overestimation of the Proteobacteria, Actinobacteria, Bacteroidetes and Chlorobi phyla; and by an underestimation of the Acidobacteria, Firmicutes, Planctomycetes and Spirochaetes phyla. According to these observations, Metaxa2-SILVA combination presented the closest expected abundance of the Spirochaetes phylum but also the most abundant false-positive rate for the not expected phyla category (Fig. 5C). On the other hand, the method with the lowest false positive rate was Metaxa2-GG with only a few bad annotations to Tenericutes and Nistropinae phyla. The rest of the methods had from 0.01 to 0.1% of annotation to Other phyla category in total relative abundance, pointing to a variety of 23 different phyla.

We observed some opposite biases between BLAST-based and -independent methods, for a given phylum, while using WMS data. In the case of Proteobacteria and Acidobacteria phyla, their abundances were underestimated by most of the BLAST-independent methods (Fig. 5D), while BLAST-dependent methods (Fig. 5C) overestimated them. Other phyla like Firmicutes and Spirochaetes were underestimated by most methods using WMS data, but in different magnitude. On the other hand, when comparing k-mers spectra methods to those using single copy marker genes (SCMG) for taxonomic assignation, we observed in the later a greater tendency to overestimate Chloroflexi, Chlorobi, Verrucomicrobia, and Crenarchaeota phyla (Fig. 5D).

## Discussion

A well-known disadvantage of using 16S rRNA genes or its variables regions as phylogenetic marker is the similarity of sequences between non-closely related species^41,42^. Our datasets were constructed from reference genomes of isolated strains, so the presence of identical sequences from different organisms, could happen in real samples. After a clustering at 100% of identity, we observed that ~27–29% of the genomes had an identical V3-V4 region. Notably, the most of these clusters were formed from genomes of the same species (~16.5% of ~27–29%) or genera (~8.5%); and only a small proportion contained genomes from different families (~3.20%) or classes (~0.3%) (https://github.com/Ales-ibt/Metagenomic-benchmark/tree/master/datasets_16SrRNA/clustering). This means that the lack of resolution at species level of the V3-V4 variable regions of the 16S rRNA phylogenetic marker is a biological issue. The methods tested in this work could either classify these sequences correctly (TP), don’t classify it (FN) or classify them wrongly (FP).

On the one hand, there are those algorithms which classify at the lower taxonomic levels when they find ambiguity in upper levels, reporting the LCA (Metaxa2 or SPINGO). In this case, the methods compromise their sensitivity at genus, species or subspecies level. On the other hand, the methods that use one of the best alignment hits to classify ambiguities (as Parallel-meta), were affected in the specificity, since they risk reporting an incorrect assignment. The chance of a correct assignment at a given taxonomic level will decrease according to the number of identical sequences in the database.

The Parallel-meta-MTX combination presented the best results among BLAST-alignment based methods for 16S rRNA amplicon dataset analysis, overperforming the Metaxa2 algorithm. An important difference between Parallel-meta ad Metaxa2 is that the former use by default megablast settings (a bigger word size, different match/mismatch scores and gap penalties), while the latter use the default blastn settings. According to this, we expected an improvement in the Metaxa2 assignments using the megablast option. A mini-test revealed that performance statistical descriptors was almost identical, indicating that the differences observed between Parallel-meta and Metaxa2 are independent of the blast parameters and can be attributed completely to the algorithm (find the results and a detailed discussion in https://github.com/Ales-ibt/Metagenomic-benchmark/Metaxa2_blast_megablast.txt). Parallel-meta reports the best hit from the Blast search, while Metaxa2, among other things, performs a filter based on its reliability score.

Until class taxonomic level, Metaxa2 and Parallel-meta (both using MTX database) had a similar performance, but a notably difference was observed between order and genus levels. However, the sensitivity gap between these tools can be reduced at a cost of a higher error rate. Higher sensitivity tends to present a lower specificity and vice versa (Figs 1A–C and 2A,B) which is a well-known trade-off between those measures.

For BLAST-independent methods using amplicon data, QIIME performed better than SPINGO at almost every taxonomic level regardless the database combination. Notably, in terms of accuracy at species level, SPINGO-RDP performed better than any other method (Fig. 1D–F). This is consistent to the results reported by SPINGO authors, but based on MCC score values, the method had a poor performance (Fig. 6A). This is a good example of the convenience of using MCC values, which weight all four possible classes (TP, FP, TN, FN) in a confusion matrix.

The database effect was observed at different levels regardless the method. In terms of sensitivity, the MTX database had a positive effect in every method and taxonomic level, even at more stringent error rate cut-off (Fig. 1D–F). For specificity, QIIME-RDP was better at any combination and taxonomic level (Fig. 2D). However, MTX database increased the accuracy of Parallel-meta and QIIME at every taxonomic level (Fig. 2C). A foreseen problem of using the MTX database for taxonomic annotation is the lack of maintenance. Currently, it is a well curated database, but as far as we know remains static. Therefore, the sensitivity of any method will be affected unless new information is added to this database.

The QIIME algorithm has a clustering step where only a representative of each cluster is used for the taxonomic assignation, reducing the possible number of true positives and false negatives (higher specificity). Conversely, BLAST-alignment based methods annotate every sequence, increasing both true positive and false positive rates. However, the positive and negative rates could be controlled by using more strict cut-off values, improving the method performance (see Supplementary Table 2).

In the overall performance according to the MCC evaluation, we observed that QIIME-RDP is the best combination for results between superkingdom and class taxonomic levels. At order and family levels, QIIME-MTX gave better results (Fig. 6A). The Parallel-meta-MTX combination performed particularly better at genus and species levels. Also, SPINGO-MTX was the best combination at those taxonomic levels, confirming the positive effect of the MTX at lower taxonomic ranks. Interestingly, the popular GG database (set as default in the QIIME pipeline), did not improved the results of any evaluated method. Databases such as SILVA and GG in combination with any method, presented MCC values below 0.5 at all taxonomic ranks and at species and subspecies levels, they presented MCC negative values (Fig. 6A) indicating a performance worse than random assignment. In particular, SPINGO which relies on a k-mer spectra algorithm, was the most affected in combination with SILVA, probably due to misleading k-mer information. Smaller but highly curated databases such as RDP and MTX improved the overall performance of all methods at almost every taxonomic level, suggesting a positive effect related to the database size and curation refinement. However, at species and subspecies level, all methods presented MCC values close to zero (Fig. 6A), suggesting that annotations at these taxonomic levels is not reliable using 16S rRNA (V3-V4 regions) amplicon sequencing.

The datasets included a portion of shuffled sequences (Material and Methods) that increased the false positive rate in some method combinations, particularly for SPINGO. The use of a highly curated database such as MTX (~88,000 sequences) which is smaller than GG and RDP databases (~1 and 3 Million, respectively), resulted in a better annotation. This was clearly reflected not only on the coverage but the lower error rate observed in methods such as QIIME and Parallel-meta v2.4.1 (Fig. 6A).

We observed higher specificity and accuracy rates for all methods relying on 16S rRNA gene information extracted from WMS than from amplicon data. This trend is evident despite algorithm and technical differences between amplicon and WMS tool-database combinations (Figs 2A,B and 4A,B). We can relate the increase of specificity (and accuracy) to the availability of full 16S rRNA gene, represented by simulated short reads with a certain sequencing depth and abundance of each genome in the community. Its recovery is possible due to the use of Hidden Markov Models in the algorithms, which is a very sensitive method. For WMS data, the 16S rRNA gene represents a small fraction of the total data and it depicts the universe of assignable reads. On the other hand, for amplicon sequencing the higher sensitivity (Supplementary Figs 1 and 3), can be related to the genome representability in the amplicon dataset. Nevertheless, all methods showed better MCC values with WMS data than amplicon reads, with scores near to 0.25 (Fig. 6A,B).

Methods based on SCMG or k-mer spectra annotation presented the highest coverage at every taxonomic level. In particular, we observed that MetaPhlAn2 and Kraken had the highest coverage at any error rate or taxonomic level, except at species rank and 1% of error cut-off where CLARK showed the highest coverage (Fig. 3D). Kraken and MOCAT were the most accurate and specific methods, with small differences (at order and genus levels) (Fig. 4C,D). We observed again the classic trade-off between sensitivity and specificity for CLARK and MetaPhlAn2, specially at species and subspecies levels. However, MetaPhlAn2 performed very well at subspecies level, even better than the best BLAST-alignment based combination, Parallel-meta-MTX (Fig. 6B).

Interestingly, CLARK accuracy drop was due to taxonomic assignment errors involving shuffled sequences. This was not the case for Kraken, which can filter information by using a last common ancestor k-mer weighting assignment algorithm, reducing the false positive rate.

Databases created from reference genomes gave a better classification as seen with Kraken and single copy gene marker methods, when comparing to 16S rRNA marker gene databases (see Fig. 6B), especially when it is enriched in sequences of organisms that have not been properly characterized (i.e. SILVA). Moreover, the more genetic information, the more accurate the taxonomic classification is.

Several metagenomic studies report results and compare environments using high taxonomic levels such as phylum. However, in this study we report that abundance biases can be observed even at such high rank. BLAST-alignment based methods presented a higher bias when combined with large databases such as GG and SILVA but only for a few phyla. Regarding the BLAST-independent methods, SPINGO in combination with almost all databases, under or overestimated the abundance of 28 different phyla but in low rates. These errors represent a greater bias than observed for BLAST-based methods, which presented a higher proportion of false positives but distributed in only four different phyla (Fig. 5A and Supplementary Table 2). The QIIME and Parallel-meta methods in combination with MTX database did not assign any of the amplicon shuffled sequences which is reflected in all the tested metrics, presenting a very good balanced performance. Conversely, Metaxa2 and SPINGO assigned different numbers of shuffled sequences regardless the database used. Notably, most of these false positives were annotated with assignment scores lower than those for true positives, what making filtering easy.

The observed results from 16S rRNA assignment methods using WMS data, presented a more distributed bias among several phyla. It should be noted that in the tested datasets, Spirochaetes phylum contains information from an *in silico* modified *Leptospira interrogans* genome, where sequences from this non-existent relative were present. In particular, most of the method combinations assigned incorrectly those sequences to lower taxonomic levels except for Metaxa2-SILVA one. Nonetheless, Metaxa2-SILVA had the most abundant false-positive rate to not expected phyla (Fig. 5C), all of them pointing to an unidentified_marine_bacterioplankton. The main difference between SILVA and the rest of the databases is that it contains sequences from uncultured, poorly characterized bacteria, that increase the likelihood of reporting an erroneous hit, raising the false positive rates no matter the method.

Despite the presence of shuffled sequences, these data did not generate a significant bias for BLAST-alignment based methods.

A non-systematic bias was observed in the results generated from WMS data. The false positive rate was distributed randomly among a couple of phyla. However, CLARK presented a higher false positive rate distributed in several not expected phyla, resulting in a higher abundance bias (Fig. 5D). Our results differ from those reported by CLARK authors, although their datasets focused on other variables such as sequencing platform error rates and their metrics were calculated differently.

We observed methods presented different biases: 1) erroneous abundance assignment, where a few phyla were largely over or underrepresented or 2) erroneous richness assignment where several phyla were artificially reported. These two errors could impact the microbial diversity interpretation in any metagenomic project.

Most of the methods are not contemporary and have not been evaluated altogether using the same dataset and database, which increases the challenge to compare their performance. Also, databases have grown and suffered changes in the taxonomic annotation that can have a direct impact in the results of any project. Therefore, we have developed an eclectic benchmarking framework to compare objectively the selected tools.

For taxonomic annotation methods based on data from 16S rRNA sequencing, the latest publication corresponds to Parallel-meta 3 and Metaxa2^31,43^ where the comparison to other tools was not extensive. While Metaxa2 authors explored the effect of databases and sequencing approaches (amplicons and WMS), Parallel-meta developers focused on the speed of their software. However, even if both methods performed better in comparison to QIIME at genus, species and subspecies levels, the datasets and evaluation criteria were different. Our results are consistent with the comparison made by the cited authors, but for classification at higher taxonomic levels, we observed that QIIME-RDP could be a better option (Table 1).

**Table 1:** Performance descriptors for the best methods ranked according to MCC at every taxonomic level.

Taxonomic level	16S rRNA amplicon					Whole Metagenome Shotgun				
	Method	MCC	ACC	Spec^a^	Sens^b^	Method	MCC	ACC	Spec^a^	Sens^b^
Phylum	QIIME-RDP	1.000	1.000	1.000	1.000	Parallel-meta-GG	0.997	1.000	1.000	0.999
Class	Metaxa2-MTX	0.961	0.996	0.944	0.999	MOCAT	0.991	0.997	0.996	1.000
Order	QIIME-MTX	0.824	0.98	0.753	0.996	MOCAT	0.990	0.997	0.996	1.000
Family	QIIME-MTX	0.553	0.916	0.374	0.995	MOCAT	0.974	0.992	0.99	1.000
Genus	Parallel-meta-MTX	0.607	0.928	0.399	1.000	MOCAT	0.885	0.966	0.959	1.000
Species	Parallel-meta-MTX	0.083	0.661	0.147	0.908	MOCAT	0.824	0.948	0.940	1.000
Subspecies	SPINGO-MTX	0.061	0.249	0.898	0.213	MetaPhlAn2	0.546	0.736	0.947	0.64

^a^Specificity; ^b^Sensitivity.

SPINGO is a novel method based on k-mer annotation designed to annotate sequences at genus or species level. If a query sequence contains a set of k-mers associated to more than one reference, the method labels it as AMBIGUOUS and reports a list of possible matches. Here, we manually curated the ambiguous results to report the LCA. Consistently with the results reported by Allard *et al*.^30^, we found that SPINGO-RDP was the method with the highest accuracy at the species level (Fig. 2C). Also, we found that this method presented the lowest specificity regardless the database combination (Fig. 2D). However, the AMBIGUOUS classification could be very convenient and easy to filter from the reported results. For our datasets, the taxonomic annotation for 25 to 35% of shuffled sequences were assigned as AMBIGUOUS but with a lower similarity score than for true positives. This indicates that the score similarity is a better criterion for filtering false positives than the AMBIGUOUS label. Only SPINGO-SILVA reported annotation for shuffled sequences with scores closer to true positives.

A recent comprehensive benchmark^20^ evaluated eleven tools and combinations between them, to classify WMS data. This study is probably the most extensive to date, where in addition to the performance of each tool, the synergy of their combinations was analyzed. However, other sequencing approaches like amplicon target sequencing or the use of different databases, were not considered. Here, we evaluated methods based on k-mer spectra annotation and found that our results were very similar to those obtained by Ounit *et al*.^26^ in terms of coverage (equivalent to precision in the cited work) and sensitivity at genus level. Consistently with our results, CLARK overperformed Kraken at subspecies taxonomic level, as observed in Fig. 4D. However, CLARK presented a higher false positive rate mainly because of the assignment of a large number of shuffled sequences with similar assignment scores to those of the true positives, which makes filtering impossible.

On the other hand, in agreement with Truong *et al*.^27^ results, we observed that MetaPhlAn2 annotated fewer false positives and false negatives than Kraken, but the latter presented higher accuracy and specificity until species level (Fig. 4C,D). Interestingly, at subspecies level MetaPhlAn2 overperformed Kraken in specificity as is shown in Fig. 4D.

As far as we know, our study is the first to benchmark MOCAT against other bioinformatics tools. Kultima *et al*.^24^ reports a high agreement between expected diversity and MOCAT annotations at genus level for real and simulated datasets. According to our results and based on MCC values, MOCAT is the best annotation method for class to species ranks (Table 1). Its higher specificity can be related to its very low false positive rate. Methods based on SCMG annotation presented better performance than any other evaluated method (Table 1). Even though their databases are smaller in size in comparison to either 16S rRNA or whole genome databases, the redundant information provided by several markers solve the lack of resolution or sensitivity for certain taxonomic groups.

## Conclusions

To the extent of our knowledge, this is the first study where several tools, developed in the last decade, are compared using a standard methodology with coverage, error rate and statistical measures of classification We can relate those metrics to scores and set a cut-off line for each method, seeking for higher sensitivity or specificity. Depending on the goals, sensitivity or specificity rates could have a different impact in metagenomic projects. Our results indicate that Parallel-meta-MTX combination is the best option for the analysis of the V3-V4 16S rRNA region at genus level, bearing in mind that at species and subspecies ranks, it will present higher error rate and lower sensitivity. Smaller but highly curated databases like RDP and MTX improved the results of tested methods in terms of sensitivity, specificity and accuracy. The standardization of taxonomic lineage is necessary to compare results, especially when the annotation was performed using different databases.

The overall performance of almost all methods using WMS data was better, but with an expected trade-off cost between sensitivity and specificity. High accuracy at low taxonomic levels could be convenient for a metagenomic project, especially if species or subspecies characterization is a relevant goal. However, is important to consider some problems for WMS sequencing approaches. Extraction of DNA at concentration and molecular weight from metagenomic samples, could be a challenge but necessary for amplification-free sequencing libraries. Also, if not all genomes present in the studied metagenomes were present in the reference database, which is the common case in environmental samples, the 16S-based methods would probably perform better than the WMS ones, as 16S rRNA databases are much extensive. However, several metagenomic studies are delivering hundreds or thousands of complete and draft bacterial genomes which will improve genome databases and WMS-based classification methods^44–47^. Finally, our work is delimited to bacterial and archaea taxonomy classification but in real life samples, the presence of eukaryotes could contribute to other misclassification problems that are not considered in our benchmark. These problems include the amplification and misclassification of ribosomal sequences belonging to mitochondrial or chloroplast genomes.

The results presented here could help other researchers to choose among the available tools, being aware of their advantages and disadvantages. Also, benchmarking of new tools could be done following our standard framework if the evaluated method reports a score for each assignment. Detailed information to benchmark, evaluate and choose the best of the tested tools, can be found at https://github.com/Ales-ibt/Metagenomic-benchmark. While this benchmark suite may be useful and available for reproducibility and implementation, is not free from the same problems of database dependence, manually defined criteria and software changes. Finally, we would like to highlight the importance of gold standards, recurrent evaluation of tools, databases curation and manual inspection of the taxonomic profiling results, for a better and more accurate microbial diversity description.

## Electronic supplementary material

Supplementary Material

Supplementary Table 2
