# Author Correction: Analysis of sequencing strategies and tools for taxonomic annotation: Defining standards for progressive metagenomics

**DOI:** 10.1038/s41598-020-61219-4

**Published:** 2020-03-03

**Authors:** Alejandra Escobar-Zepeda, Elizabeth Ernestina Godoy-Lozano, Luciana Raggi, Lorenzo Segovia, Enrique Merino, Rosa María Gutiérrez-Rios, Katy Juarez, Alexei F. Licea-Navarro, Liliana Pardo-Lopez, Alejandro Sanchez-Flores

**Affiliations:** 1 Consorcio de Investigación del Golfo de México (CIGOM), Instituto de Biotecnología, Universidad Nacional Autónoma de México, Cuernvaca, Mexico; 2 Instituto de Biotecnología, Universidad Nacional Autónoma de México, Cuernvaca, Mexico; 3 Departamento de Innovación Biomédica, CICESE. Carretera Ensenada-Tijuana 3918, Zona Playitas, Ensenada, BC Mexico

Correction to: *Scientific Reports* 10.1038/s41598-018-30515-5, published online 13 August 2018

The website on which the data underlying the study reported in Reference 17 was stored is no longer maintained. As such, in the Materials and Methods section, under subheading ‘In silico datasets for WMS and 16S rRNA profiling’,

“Sequences can be found at http://www.ucbioinformatics.org/metabenchmark.html.”

should read:

“Sequences can be found at https://zenodo.org/record/3662895#.XkMnZnUzbRI.”

