# Supplementary material for: Analysis of sequencing strategies and tools for taxonomic annotation: Defining standards for progressive metagenomics
Source: Sci Rep. 2018 Aug 13;8:12034. doi: 10.1038/s41598-018-30515-5 (PMC6089906; doi:10.1038/s41598-018-30515-5)

Analysis of sequencing strategies and tools for taxonomic annotation: Defining standards for progressive metagenomics

Escobar-Zepeda Alejandra[1+], Godoy-Lozano E. Ernestina[1+], Raggi Luciana[1+], Segovia Lorenzo[1,2], Merino Enrique[1,2], Gutierrez-Rios Rosa-Maria[1,2], Juarez Katy[1,2], Licea-Navarro Alexei F.[1,3], Pardo-Lopez Liliana[1,2] and Sanchez-Flores Alejandro[1,2*].

[1] Consorcio de Investigación del Golfo de México (CIGOM)

[2] Instituto de Biotecnología, Universidad Nacional Autónoma de México.

[3] Departamento de Innovación Biomédica. CICESE. Carretera Ensenada-Tijuana 3918, Zona Playitas, Ensenada, BC, México

[+]These authors contributed equally to this work.

*Correspondence to: alexsf@ibt.unam.mx

Supplementary information

Suppl. Table 1. List of bioinformatic tools used in this work

Software	Input data	Annotation strategy	Databases used	URL	Ref.
**Metaxa2 v2.1.1**	WMS, 16S	SSU extraction by HMMER; BLAST+ for annotation	MTX, RDP v11.5, SILVA v128 and GG v13.5	http://microbiology.se/software/metaxa2	(Bengtsson-Palme et al., 2015)
**Parallel-meta v2.4.1***	WMS, 16S	HMMER for 16S/18S extraction; MegaBLAST for annotation	RDP, GG, SILVA (included in Parallel-meta distribution) and MTX	https://github.com/Comp-Bio-Group/Parallel-META	(Su et al., 2014)
**QIIME v1.9.1**	16S	Uclust clusterization at 97% and representative sequence annotation	MTX, RDP v11.5, SILVA v128 and GG v13.5	http://qiime.org/	(Caporaso et al., 2010)
**SPINGO v1.3**	16S	K-mer comparison against a database	MTX, RDP v11.5, SILVA v128 and GG v13.5	https://github.com/GuyAllard/SPINGO	(Allard et al., 2015)
**MetaPhlAn2 v2.2.0**	WMS	Bowtie2 mapping and normalization according to the descendant's assignments	Its own database made from a set of single copy marker genes from reference genomes	http://segatalab.cibio.unitn.it/tools/metaphlan2/	(Truong et al., 2015)
**MOCAT v1.3**	WMS	SOAPaligner mapping and coverage normalization	mOTU and RefMG single copy marker genes databases	http://mocat.embl.de/index.html	(Kultima et al., 2012)
**Kraken v0.10.5-beta**	WMS	Exact k-mer mapping to LCA	k-mer profiles from RefSeq genomes	http://ccb.jhu.edu/software/kraken/	(Wood & Salzberg, 2014)
**CLARK v1.2.3.1**	WMS	Reads assignment to a reference genome by k-mer spectra comparison	k-mer profiles from RefSeq genomes	http://clark.cs.ucr.edu/Tool/	(Ounit & Lonardi, 2016)

Suppl. Figure 1. 16S amplicon BLAST-based methods sensitivity

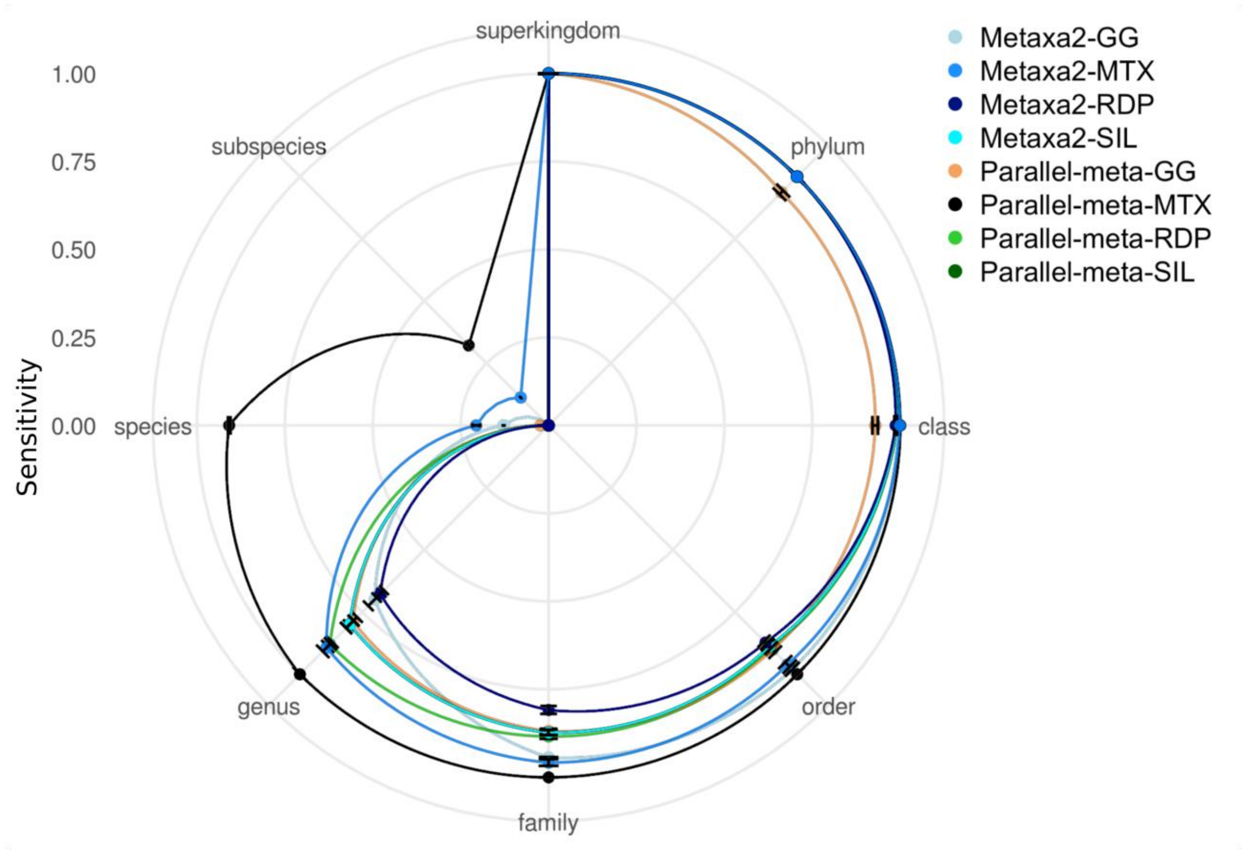

Suppl. Figure 2. 16S amplicon BLAST-independent methods sensitivity

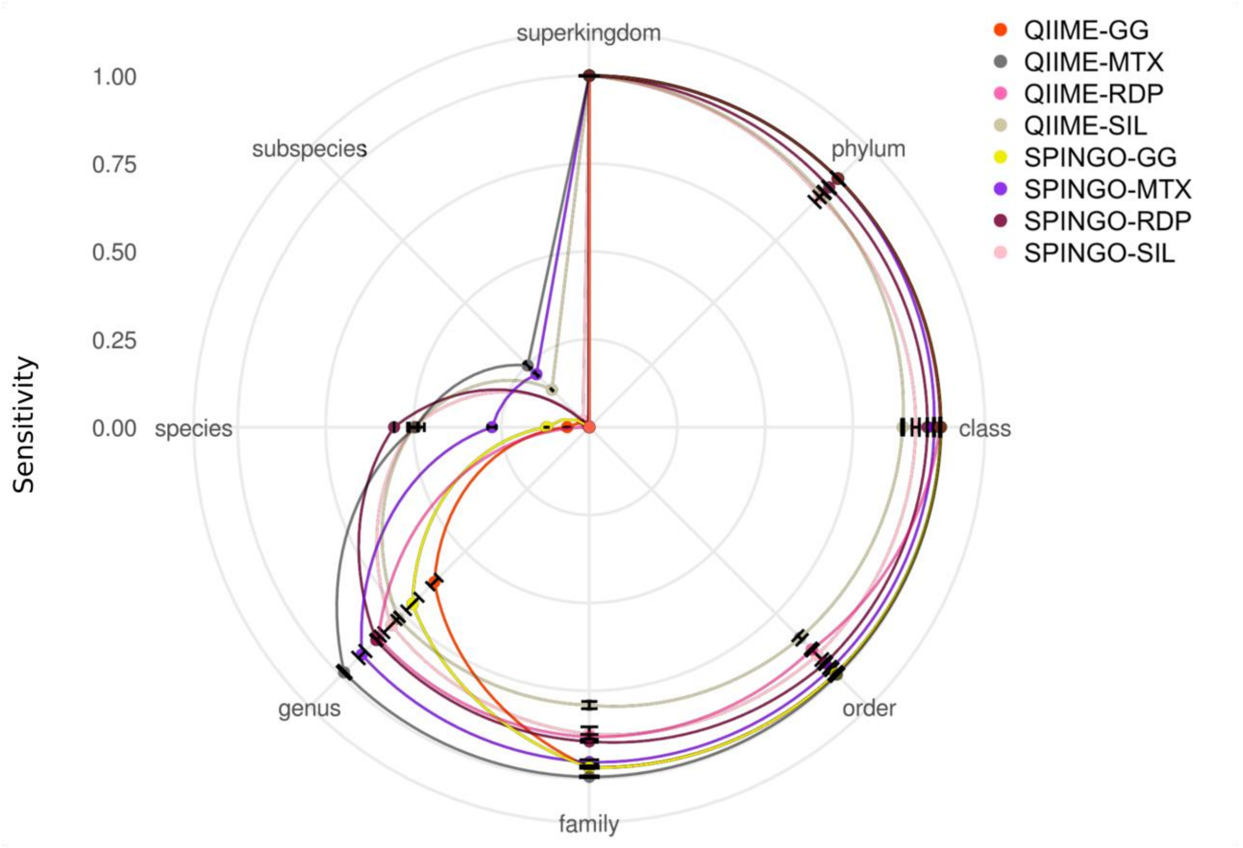

Suppl. Figure 3. WMS data BLAST-based methods sensitivity

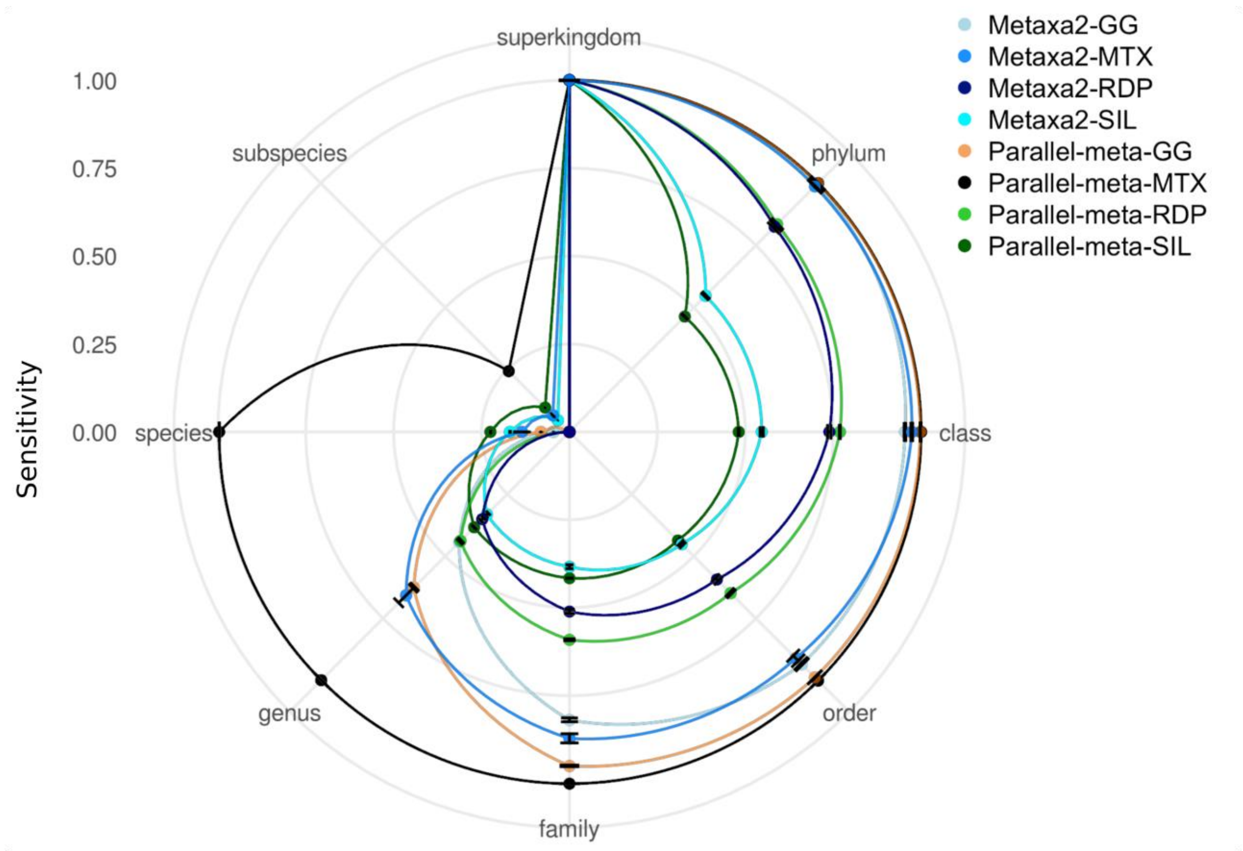

Suppl. Figure 4. WMS data BLAST-independent methods sensitivity

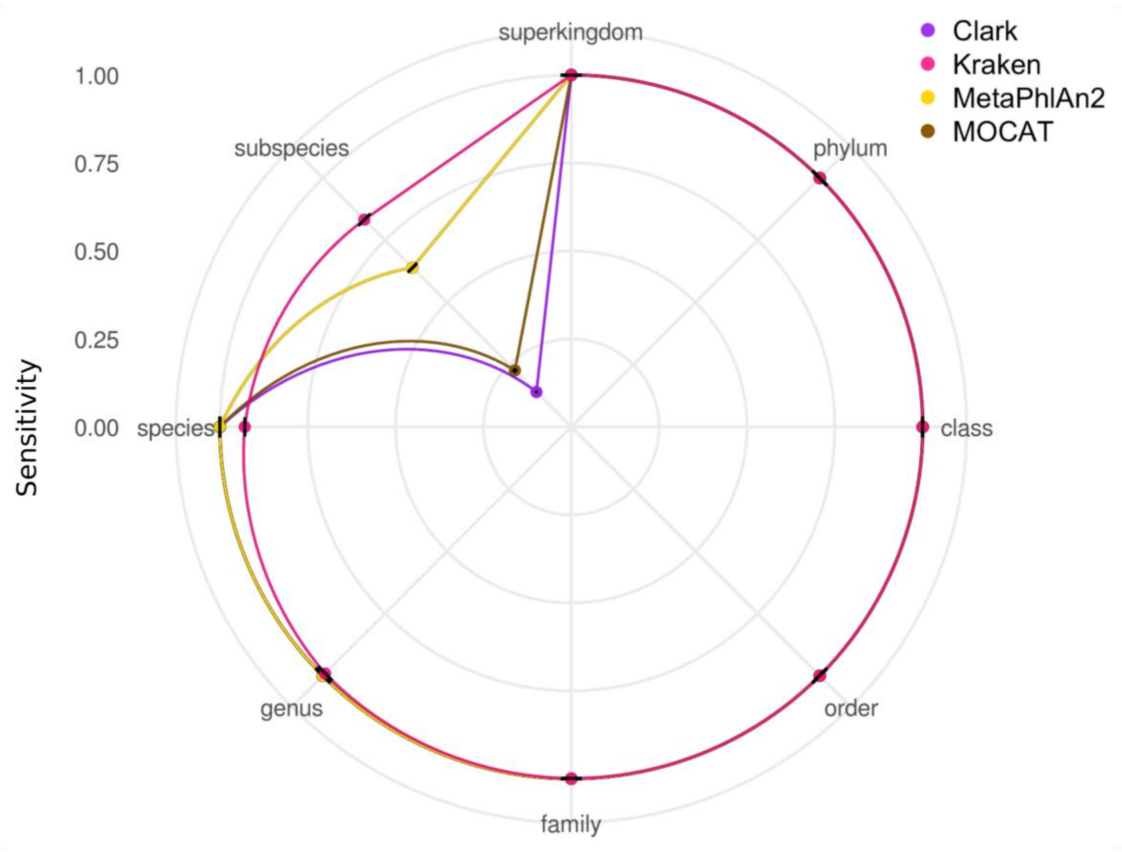

Supplement: Supplementary file 1 — Supplementary Material [file 41598_2018_30515_MOESM1_ESM.pdf]
